# Space-time heterogeneity of hand, foot and mouth disease in children and its potential driving factors in Henan, China

**DOI:** 10.1186/s12879-018-3546-2

**Published:** 2018-12-07

**Authors:** Xiangxue Zhang, Chengdong Xu, Gexin Xiao

**Affiliations:** 1 The School of Earth Science and Resources, Chang’an University, Xi’an, 710054 China; 2 State Key Laboratory of Resources and Environmental Information System, Institute of Geographic Sciences and Natural Resources Research, Chinese Academy of Sciences, 11A, Datun Road, Chaoyang District, Beijing, 100101 China; 3 China National Center for Food Safety Risk Assessment, Beijing, 100022 China

**Keywords:** Hand, Foot, and mouth disease, Meteorological factors, Socio-economic factors, Spatiotemporal risk, Bayesian space-time hierarchy model

## Abstract

**Background:**

Hand, foot and mouth disease (HFMD) has become a substantial threat recently. However few studies have quantified spatiotemporal heterogeneity of HFMD and detected spatiotemporal interactive effect of potential driving factors on this disease.

**Methods:**

Using GeoDetector and Bayesian space-time hierarchy model, we characterized the epidemiology of HFMD in Henan, one of the largest population provinces in China, from 2012 to 2013, and quantified the impacts of potential driving factors.

**Results:**

Notably, 21.43 and 24.60% counties were identified as hot and cold spots, respectively. Spatially, the hotspots were mainly clustered in regions where the economic level was high. Temporally, the highest incidence period of HFMD was discovered to be in late spring and early summer. The impact of meteorological and socio-economic factors on the disease are significant, and this study found that a 1 °C rise in temperature was related to an increase of 4.09% in the HFMD incidence, a 1% increment in relative humidity was associated with a 1.77% increase of the disease, and a 1% increment in ratio of urban to rural population was associated with a 0.16% increase of the disease.

**Conclusion:**

Meteorological and socio-economic factors presented significantly association with HFMD incidence, high-risk mainly appeared in large cities and their adjacent regions in hot and humid season. These findings will be helpful for HFMD risk control and disease-prevention policies implementation.

**Electronic supplementary material:**

The online version of this article (10.1186/s12879-018-3546-2) contains supplementary material, which is available to authorized users.

## Background

Hand, foot and mouth disease (HFMD) is a worldwide infectious disease [1]. It is mainly caused by Coxsackie virus A16 (CV-A16) or enterovirus 71 (EV71) [2–4]. This disease is characterized by flu-like clinical symptoms including fever, mouth ulcers, poor appetite, vomiting, diarrhea, and rashes on the hands, feet, and buttocks [2, 4]. It is believed to be transmitted mainly through direct contact with contaminated discharges, contaminated objects, and fluid from blisters or stool from infected persons, with an average incubation period of three to 7 days [5, 6]. This disease continues to be a serious public health threat, especially to children, as there is no definitive treatment for HFMD, currently.

During the past decades, HFMD outbreak has occurred in numerous areas, especially in the Asia-Pacific region, such as Thailand [7], Taiwan [8], Singapore [9], Hong Kong [10], Vietnam [11], Malaysia [12], Japan [13] and parts of mainland China [14, 15]. In 2007 and early 2008, mainland China experienced several serious outbreaks of HFMD and established a national enhanced surveillance system to respond those outbreaks [15]. In May 2008, HFMD was defined as a Class C infectious disease that requires reporting of every case [16]. A considerable threat still exists, because HFMD especially affects areas of high economic level, possesses distinctive seasonality, and can result in death in severe cases.

Some studies have determined that HFMD risk has temporal variations. It is well accepted that meteorological factors play an important role in the transmission of HFMD. For example, in Finland and Japan, a single season peak of HFMD has been observed during the summer and early autumn months, separately [13, 17]. Meanwhile, an annual peaks in the warmer months (May to July) and a smaller winter peak (October to December) have been detected in subtropical and tropical regions, including Hong Kong, Malaysia, and parts of mainland China [6, 10, 12, 18, 19]. Furthermore, the annual peak of incidence seasonality has varied from April in the southern area to July in the northern area of China [15]. In recent years, there has been increased interest in exploring the impact of meteorological factors on HFMD, such as temperature [20–23], relative humidity [20, 23], precipitation [15, 20, 22], wind speed [15, 20], hours of sunlight [15], and air pressure [15, 21].

Meanwhile, the risk of HFMD also presents obvious spatial heterogeneity. Some studies indicated that it was closely correlated to socio-economic variables: demographics, local geographic environment, socio-economic status, health conditions, and infrastructure. For example, Yan et al. showed that HFMD incidence was higher in urban areas compared with rural areas and demonstrated that the distance to the nearest freeway and per capital GDP are risk factors associated with HFMD incidence [24]. Hu et al. indicated that the population density of children can explain 56% of the variance in the cumulative monthly HFMD incidences in 2912 counties in China [23]. Likewise, rural-to-urban migrant-worker parents were found to be a major risk factor associated with HFMD in children [25], which implies that socio-economic factors also play an essential role in the transmission and spread of HFMD.

To our knowledge, few studies have quantified spatiotemporal heterogeneity of HFMD and detected spatiotemporal interactive effect of potential driving factors on this disease in the study region. The aims of this study are to 1) reveal the county-level spatiotemporal heterogeneity of HFMD risk, 2) detect the hot/cold spots, and 3) quantify the relationships between meteorological, socio-economic factors and HFMD incidence.

## Methods

### Study area

**Fig. 1:**
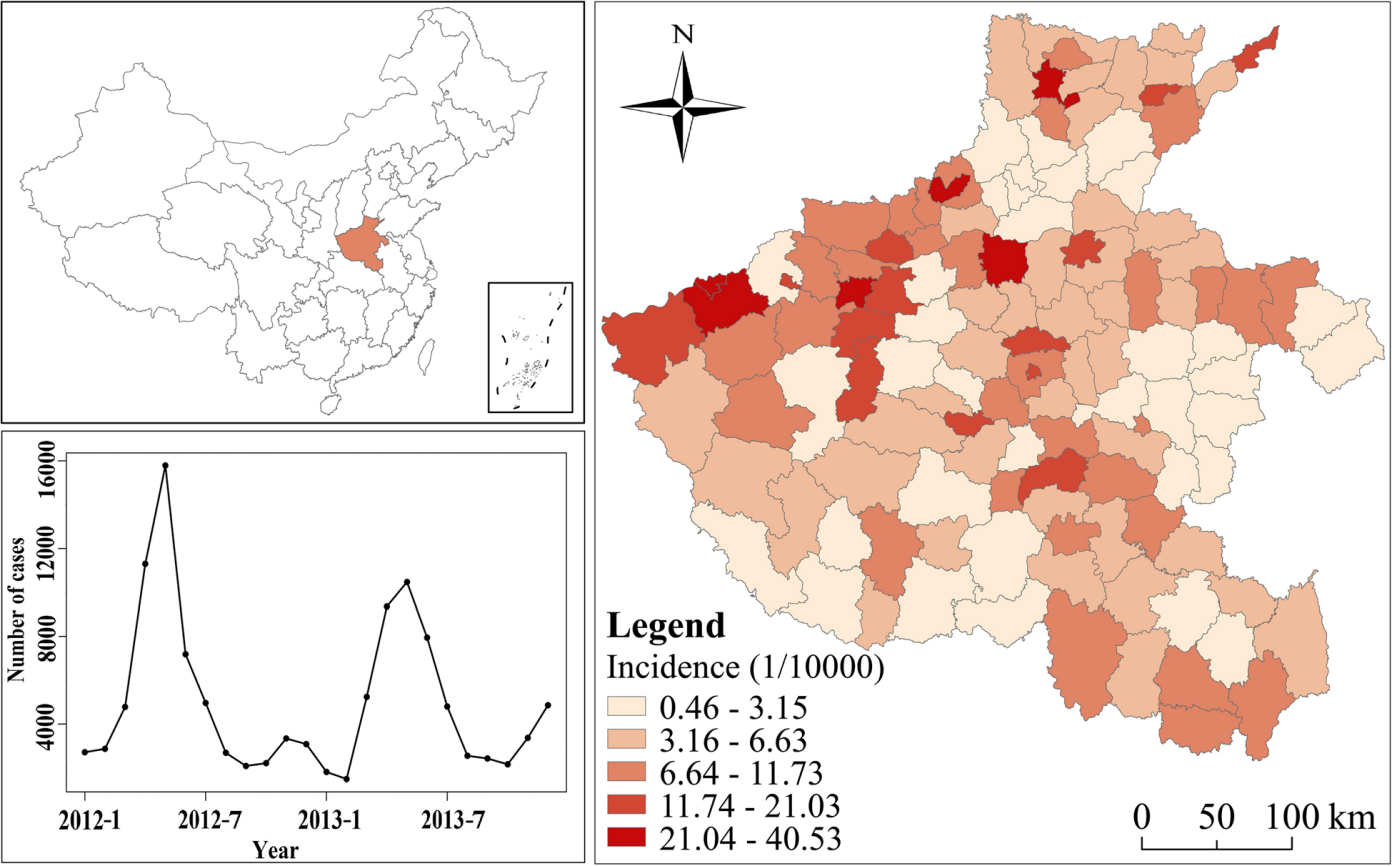
Geographic location of the Henan province in China, and cumulative monthly incidence of HFMD in children from 2012 to 2013. (The administrative map in the figure was obtained from the Resource and Environment Data Cloud Platform (http://www.resdc.cn))

Henan, as one of the provinces with the largest population and the greatest population mobility, is located in the latitude 31.23° to 36.22°N and longitude 110.21° to 116.39°E and has a population close to 95.32 million within an area of 167,000 km^2^ (Fig. 1). It includes millions of immigrants and migrants, mainly to other provinces in China. Henan has a warm and humid monsoon climate, with four distinctive seasons: a dry and windy spring, hot and humid summer, warm and sunny autumn, cold and dry winter. The average annual temperature and precipitation in the province are 15 °C and 672 mm, respectively.

### Data sources

**Fig. 2:**
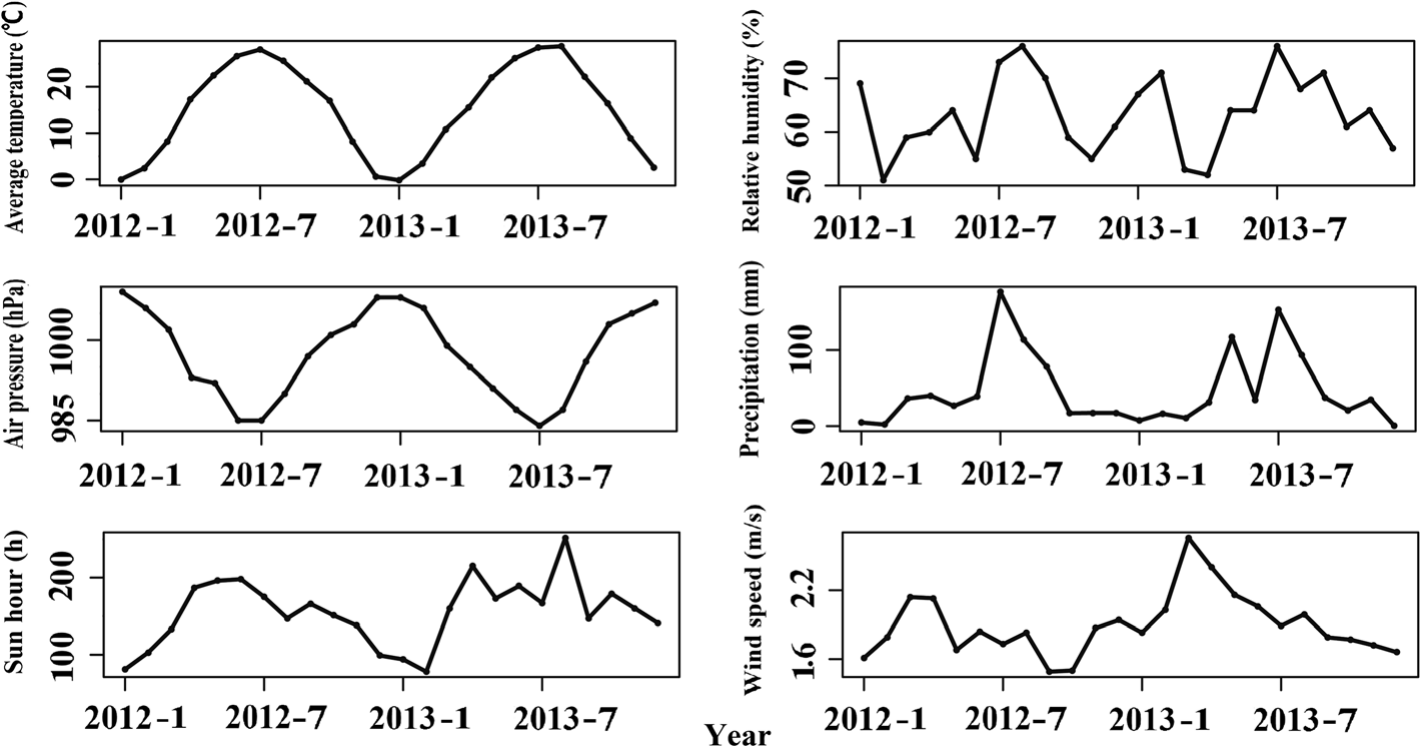
Temporal evolution in potential meteorological factors from 2012 to 2013

Data on HFMD cases from January 1, 2012 to December 31, 2013 were obtained from the Chinese Centre for Disease Control and Prevention for use in this study. Monthly meteorological data for the same period was obtained from the China Meteorological Data Sharing Service System and includes average temperature, relative humidity, wind speed, precipitation, hours of sunlight, and air pressure (Fig. 2). The county level socio-economic variables from 2012 to 2013 were acquired from the governmental economic statistical yearbooks of Henan province, including the ratio of urban to rural population, population density of children under five, per capita Gross Domestic Product (GDP), per capita income of farmers, high school enrollment rate, and industrial structures (Additional file 1: Table S1). The administrative map used in the study was obtained from the Resource and Environment Data Cloud Platform (http://www.resdc.cn).

### GeoDetector

In this study, GeoDetector *q* statistic [26–28], was used to quantify the spatial and temporal stratified heterogeneity and assess their interactive affect for risks of HFMD.

The GeoDetector *q* value can be expressed as:

1 $$ q=1-\frac{1}{N{\sigma}^2}{\sum}_{h=1}^L{N}_h{\sigma}_h^2 $$

where *q* denotes the level of spatial, temporal or spatiotemporal stratified heterogeneity for target variable, e.g., HFMD risk. Its value ranges from 0 to 1, if the value approach 1 indicates the distribution of the variable has strong heterogeneity, otherwise if the value approach 0 indicates the variable has random distribution. *N* is the number of counties. *σ*^2^ and ${\sigma}_h^2$ are the variance over all the statistical units in the study area and within stratum *h (h* = 1, 2,…, *L)*, respectively.

### Bayesian space-time hierarchy model

Bayesian space-time hierarchy model (BSTHM) was used to analyze the temporal and spatial variations of disease risk. This model can explore the spatial-temporal heterogeneity of disease risk, quantify the impacts of potential driving factors, and highlight the changes in local or common trends.

The Poisson with log link regression function was used to model the data. Supposing that, in area *i* (*i* = 1, 2,…, 126) and month *t* (*t* = 1, 2,…, 24), *y*_*it*_ and *n*_*it*_ represent the number of cases and the risk population respectively, and disease cases can be described as followings:

2 $$ {\displaystyle \begin{array}{c}{y}_{it}\sim Poisson\left({n}_{it}{u}_{it}\right)\\ {}\mathit{\log}\left({u}_{it}\right)=a+{s}_i+\left({b}_0{t}^{\ast }+{v}_t\right)+{b}_{1i}{t}^{\ast }+{\sum}_{n=1}^N{\beta}_n{x}_{nit}+{\varepsilon}_{it}\end{array}} $$

where *u*_*it*_ denotes the potential risk of HFMD in region *i* and month *t*. The term *α* is the overall log disease risk during a selected period in the study region. The spatial term *s*_*i*_ indicates the disease risks in county *i*. The overall time trend is expressed by *b*_*0*_t^*^ + *v*_*t*_, and it is composed of a linear trend *b*_*0*_t^*^ with additional Gaussian noise *v*_*t*_. Time span relative to the midpoint *t*_*mid*_ over the study period is represented by t^*^ = *t* − *t*_*mid*_. The term *b*_*1i*_t^*^ allows each county to have its own trend. Specifically, *b*_*0*_ represents the overall change rate of disease risk, while, *b*_*1i*_ measures the departure from *b*_*0*_ for each county. For example, if *b*_*1i*_ is greater than 0, the local variation intensity is higher than the overall variation trend, if *b*_*1i*_ is less than 0, the local variation intensity is lower than the overall variation trend. The regression coefficient of the risk factors is *β*. The term *x*_*nit*_ is the *n-th* risk factor for area *i* and month *t*. Gaussian noise random variable is represented by *ε*_*1i*_ [29].

The Besag, York, and Mollie (BYM) spatial model was introduced to determine the prior distribution of the parameters *s*_*i*_ and *b*_*1i*_ [30]. To enhance the random effect of spatial structure in BYM, we used the conditional autoregressive (CAR) prior with a spatial adjacency matrix *W*. The CAR prior on the spatial random effect implied that adjacent counties tend to have similar disease risks.

The temporal noise *v*_*t*_ is quantified as *v*_*t*_ ∼ *N* (0, *σ*_*v*_^*2*^) and the Gaussian noise *ε*_*it*_ is expressed as *ε*_*it*_ ~ *N* (0, *σ*_*ε*_^*2*^). As suggested by Gelman [31], the prior distribution of the standard deviations (e.g., *σ*_*v*_, *σ*_*ε*_) of all the random variables in the model is determined as a strictly positive half Gaussian distribution *N*_+*∞*_ (0, 10).

According to the posterior distribution of all parameters, the spatiotemporal heterogeneity and variation of HFMD risk was quantified. Then, the following criteria was used to classify study area into hot, cold and other spots [32]. If the posterior probability *p* (exp (*s*_*i*_) > 1 | *data*) > 0.90, a county was defined as a hotspot. Conversely, a county was defined as coldspot if the posterior probability *p* (exp (*s*_*i*_) > 1 | *data*) < 0.10. The other areas were regarded as neither hot nor cold spots. Here, exp. (*s*_*i*_) represents the average disease risk (over time) in county *i* relative to *α* [33].

All parameters were calculated by WinBUGS [34], statistical software that was designed specifically for Bayesian calculation. In addition, posterior distributions of all parameters in the model were obtained through Markov chain Monte Carlo (MCMC) simulations.

### Spatial lag model

The spatial term (*s*_*i*_) in BSTHM, was largely affected by long-term stable factors compared to meteorological factors, such as local geographic environment, socio-economic conditions, topography, and medical equipment. In the study, the spatial lag model (SLM) was used to quantify the relationships between the spatial term *s*_*i*_ and socio-economic factors. It was modeled as the following formula:

3 $$ {s}_i=\rho {\boldsymbol{W}}_{s_i}+\boldsymbol{X}\boldsymbol{\beta } +\boldsymbol{\varphi} $$

where the *ρ* was the coefficient of the spatial term. Its value ranged from 0 to 1; when the value was closer to 1, the more similar the dependent variables in adjacent areas were. The spatial adjacent matrix ***W*** reflects the spatial trend of the response variable itself. The spatial regression coefficient of the explanatory variables is represented by ***β***. The explanatory variable is represented by ***X***, which includes all selected socio-economic factors. The error term is defined by ***φ***.

## Results

### Descriptive statistics

From January 2012 to December 2013, there were a total of approximately 120 thousands cases of HFMD in children in all 126 counties of Henan province. The annual incidence in 2012 and 2013 were 88.04/ 10^4^ and 78.92/ 10^4^, respectively.

**Fig. 3:**
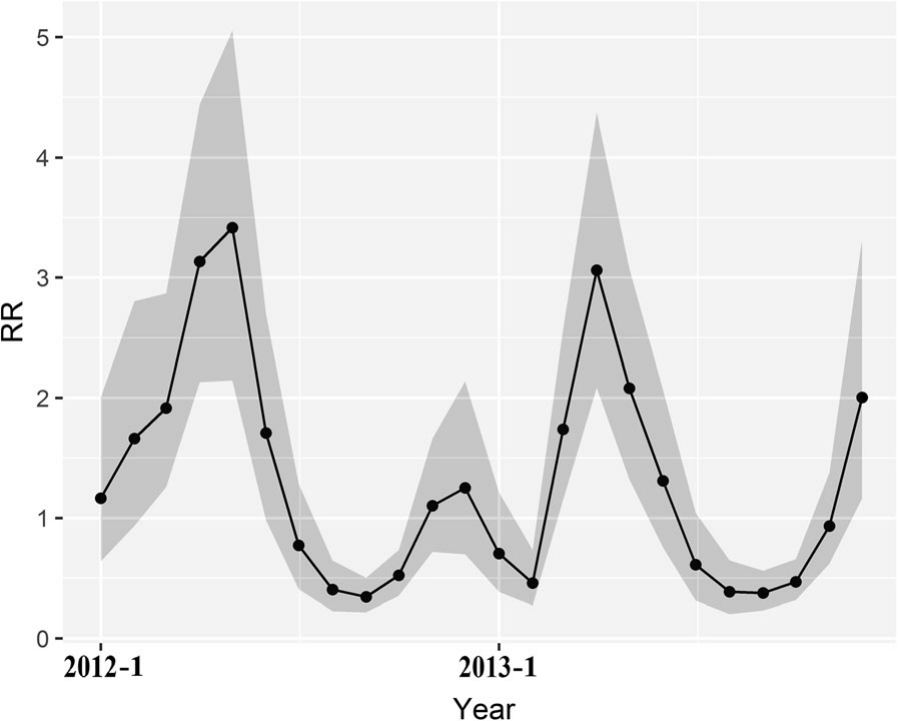
The posterior means of the temporal relative risks (exp(*b*_*0*_t^*^ + *v*_*t*_)) of HFMD in children from 2012 to 2013

Figure 3 showed the overall temporal trend of HFMD risk from 2012 to 2013, which denoted that the temporal relative risk differed significantly between months, as the GeoDetector *q* value was 0.35 (*p* < 0.01), indicating that there was an obvious seasonal variation in the risks of HFMD. The period of highest risk appeared in late spring and early summer (April to June), with an average monthly incidence of 14.45/10^4^, and the lower disease risk occurred in the fall season (August to October), with an average monthly incidence of 3.29/10^4^.

**Fig. 4:**
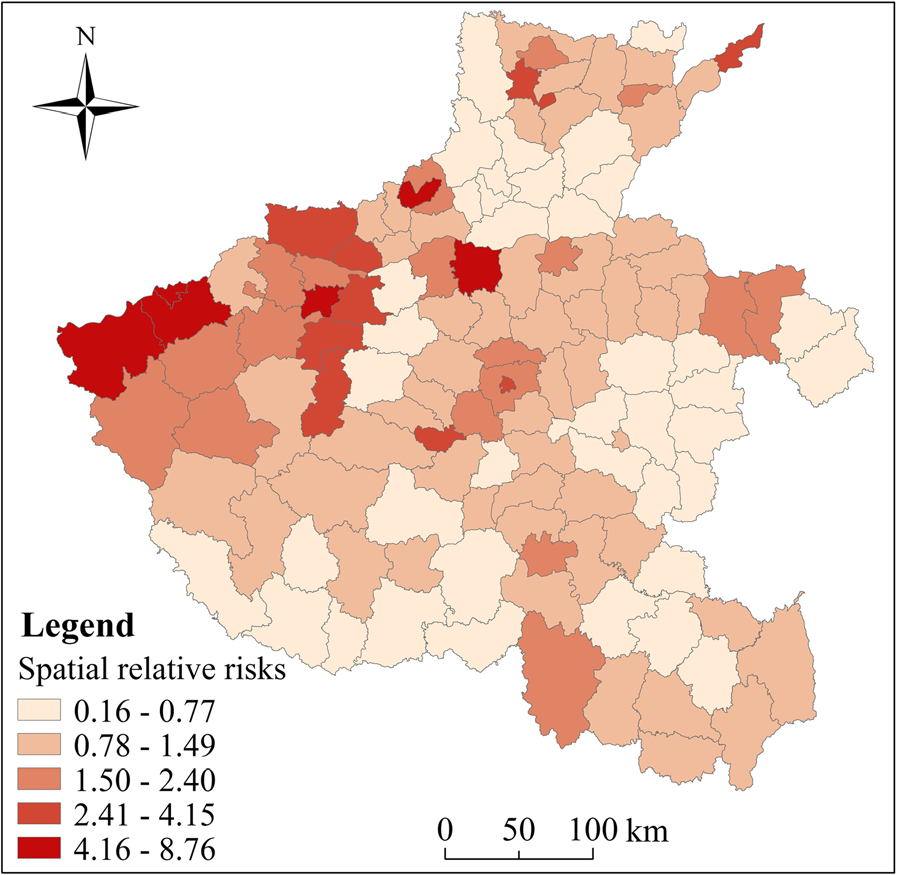
The posterior means of the spatial relative risks (RRs) (exp(*s*_*i*_)) of HFMD in children for each county, Henan province. (The administrative map in the figure was obtained from the Resource and Environment Data Cloud Platform (http://www.resdc.cn))

The relative risk (RR) of HFMD varied geographically, which indicated that there also has obvious spatial heterogeneity, as the GeoDetector *q* value was 0.31 (*p* < 0.01). Figure 4 shows the spatial RR of HFMD by county from 2012 to 2013. The high risk mainly appear in the regions where the level of economic and urbanization was high, including Zhengzhou, Jiyuan, Sanmenxia, Jiaozuo, Luoyang, Xuchang, Hebi, which was correspond to the areas where the per capita GDP was high [35].

Additionally, the spatiotemporal interaction effect of HFMD relative risk was also calculated by GeoDetector, the *q* value was 0.67 (*p* < 0.01), which indicated a significantly spatiotemporal heterogeneity.

**Fig. 5:**
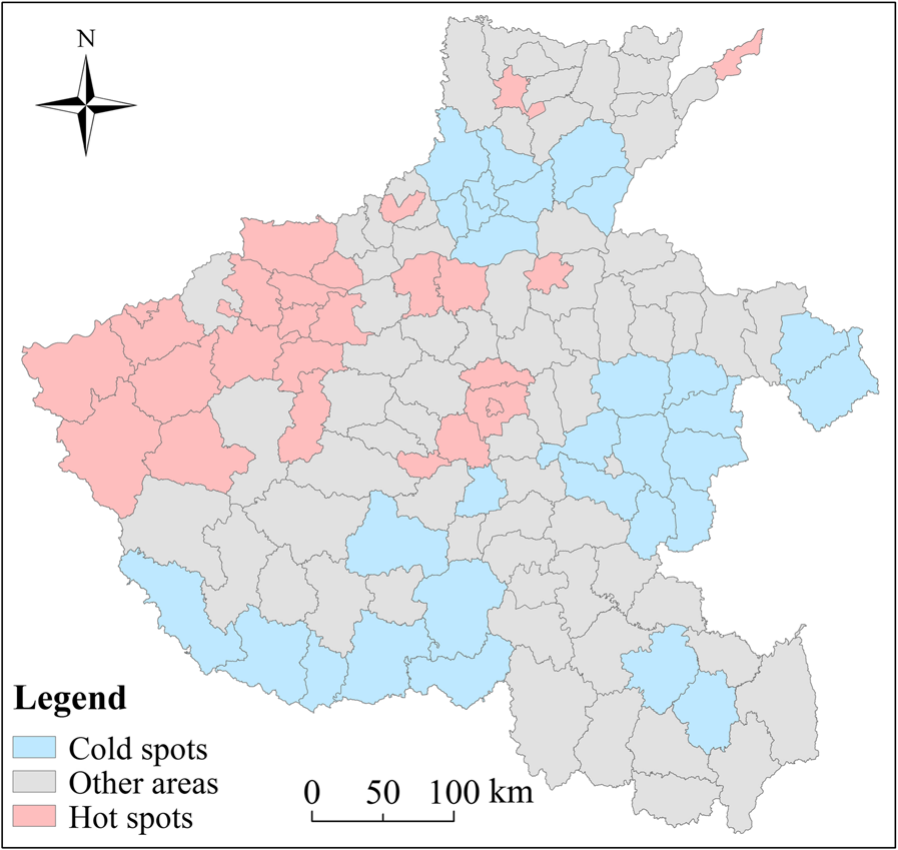
Map of the hot spots and cold spots of HFMD in each county of Henan Province. (The administrative map in the figure was obtained from the Resource and Environment Data Cloud Platform (http://www.resdc.cn))

In the study region, among the 126 counties, 27 (21.43%) and 31 (24.60%) counties were considered as hot and cold spots, respectively. Another 68 (53.97%) counties were identified as neither hot nor cold spots. Figure 5 presents that, hotspot areas were mainly distributed in economically developed areas.

To quantify the relative importance of the stable component (*s*_*i*_ + *b*_0t_* + *v*_*t*_) compared to the terms allowing for space-time interaction (*b*_*1i*_ + *ε*_*it*_) in explaining the observed space-time variation [33], we computed the posterior median of variance partition coefficient (VPC). It is the ratio of the empirical variance of (*s*_*i*_ + *b*_0t_* + *v*_*t*_) to the sum of the empirical variances of (*s*_*i*_ + *b*_0t_* + *v*_*t*_) and (*b*_*1i*_ + *ε*_*it*_) multiplied by 100%. The posterior median and 95% CI of VPC from the MCMC iterations can be obtained, which was 95.57% (with 95% CI: 93.98 to 96.74%), indicating that the stable component explained the majority of the observed variability.

### Risk factor detection

The HFMD risk showed an apparent correlation to seasonal changes (Fig. 3), and it indicated that meteorological factors played a dominant role in the temporal variation of the HFMD, in which average temperature represented the strongest influence on HFMD.

There was a positive association between average temperature and HFMD. A 1 °C rise in temperature related to an increase of 4.09% (95% CI: 1.12 to 7.27) in the risk of HFMD (RR: 1.04; 95% CI: 1.01 to 1.08) (Table 1).

**Table 1:** The quantified posterior means and RR of all coefficients in BSTHM

Meteorological factors	Posterior mean (95% CI) (100%)	RR (95% CI)
Average temperature (°C)	4.09 (1.12–7.27)	1.04 (1.01–1.08)
Relative humidity (%)	1.77 (0.68–2.77)	1.02 (1.01–1.03)
Air pressure (hPa)	0.89 (0.36–1.36)	1.01 (1.00–1.014)
Precipitation (mm)	−0.12 (− 0.23–− 0.01)	0.999 (0.998–1.00)
Sun hour (h)	−0.02 (− 0.25–0.20)	1.00 (0.998–1.002)
Wind speed (m/s)	2.21 (−13.02–16.69)	1.02 (0.88–1.18)

There was a positive association between HFMD and relative humidity. A 1% increment in relative humidity was associated with a 1.77% rise (95% CI: 0.68 to 2.77) in the risk of HFMD (RR: 1.02; 95% CI: 1.01 to 1.03) (Table 1).

A positive association was found between air pressure and HFMD. A 1 hPa increasing was related to 0.89% (95% CI: 0.36 to 1.36) rise in the HFMD risk, with corresponding RRs were 1.01 (95% CI: 1.00 to 1.014) (Table 1).

Meanwhile, precipitation was presented negative association with the HFMD risk. A 1 mm rise was linked to 0.12% (95% CI: − 0.23 to − 0.01) decrease in the HFMD risk, with corresponding RRs were 0.999 (95% CI: 0.998 to 1.00) (Table 1). Additionally, the estimated coefficients for sun hour, wind speed were not statistically significant (Table 1).

Furthermore, the risk of HFMD presented apparent spatial heterogeneity, and the study found that socio-economic factors also played a dominant role.

There was positive relationship between ratio of urban to rural population and the HFMD risk. A 1% increment in ratio of urban to rural population was associated with a 0.16% increase in the risk of HFMD (*p* < 0.01) (Table 2).

**Table 2:** The estimated coefficients of socio-economic factors in SLM

Socio-economic variables	Coefficient	Std. Error	*z*-value	*P*-value
Ratio of urban to rural population	0.16	0.05	3.51	0.00^**^
Proportion of the tertiary industry	0.02	0.01	2.24	0.03^*^
Per capita GDP	1.10	0.33	3.30	0.00^**^
Proportion of the second industry	0.02	0.01	1.73	0.08
High school penetration rate	0.002	0.01	0.28	0.78
Per capita income of farmers	−4.73	5.35	−0.88	0.38
Population density of children under five	−0.05	0.03	−1.63	0.10

^**^statistical significance level: 0.01

^*^statistical significance level: 0.05

The proportion of the tertiary industry also showed a positive association with HFMD risk. A 1% rise in proportion of the third industry may be related to an increase of 0.02% in the HFMD risk (*p* < 0.05) (Table 2).

In particular, per capita GDP presented the highest determinant power amongst these socio-economic factors. A 1000 yuan rise in per capita GDP was associated with a 1.10% increase in the HFMD risk (*p* < 0.01) (Table 2).

These results indicated a statistically significant regression relationship between the HFMD risk and the ratio of urban to rural population, proportion of the tertiary industry, and per capita GDP, as the values of *p* all are less than 0.05. There also presented on not statistically significant relationships for other selected factors (Table 2).

## Discussion

HFMD remains a serious threat to childhood health and has become one of the leading causes of childhood mortality in mainland China [15, 23, 36, 37]. In recent decades, Henan province, as one of the largest population provinces in China, has experienced several serious outbreaks of HFMD [38, 39]. The present study, from spatiotemporal perspective, explored the epidemiological characteristics of the disease, and quantified the impacts of meteorological factors and socio-economic variations on childhood HFMD incidence in Henan. The results revealed that the highest risk was mainly gathered in areas with high urbanization levels, meanwhile, meteorological factors were found have significant effects on the transmission of HFMD.

The relative risk of HFMD was linked to an obvious seasonal variation, with the highest risk appearing in late spring and early summer (April to June), and the lowest risk in autumn (August to October). It is widely accepted that meteorological factors play a decisive role in the seasonal changes of HFMD incidence, which are regarded as crucial environmental factors that influence the spread and survival of viruses causing HFMD [36, 37, 40]. The association between meteorological factors and the seasonal evolution of HFMD incidence has captured particular interests from many researchers, and some studies have reported that temperature and relative humidity played an extraordinary important role in the seasonal variation of HFMD [40–43].

The study found that average temperature was strongly positively association with monthly HFMD incidence, which is consistent with previous studies in month time scale. For example, a rise in average temperature may have led to an increase in the number of HFMD cases in Vietnam [44]. And a study showed that an increase in average temperature was associated with a rise of the number of HFMD cases [45]. The potential mechanism could be that temperature affects the behavioral patterns of people, and warmer weather can lead to increased contact, especially among young children, accordingly facilitating the spread of HFMD infection [46].

Similarly, there was a positive relationship between relative humidity and the incidence of HFMD, which is same as other studies [23, 47]. That maybe because during humid days the virus could easily attach to articles in the air, facilitating the spread of the disease [48]. However, a previous study found that relative humidity is not related to the prevalence of this disease, which was different from the present research [46].

Another important driving meteorological factor that influence the transmission of HFMD are air pressure, also presenting positive correlation with HFMD incidence, which was consistent with other study [49]. The potential mechanism may be that air pressure affects the immune system and increases the risk of disease.

Additionally, precipitation presented negative correlation with HFMD incidence, which was also consistent with other studies. Some studies demonstrated that heavy downpours could break down the survival environment of viruses [44, 50]. The potential reasons may be that precipitation would reduce social contact, thus affect the spread of the disease [51].

Furthermore, wind speed and sun hour were found to have no statistic significant association with HFMD in the study. This was consistent with some of previous studies, however, some studies have drawn opposite conclusions. For example, Liao et al. found that wind speed and sun hour has no significant association with HFMD incidence [6]. Whereas, Xiao et al. demonstrated that the weaker association presented between the sun hour and HFMD incidence [52]. Meanwhile, Wang et al. denoted that the wind speed and sun hour were found to be positively associated with HFMD [53].The potential reasons may be that these meteorological factors have different relationships with the HFMD in different regions.

These results denoted that meteorological factors play different roles in contributing to the transmission of enteric infectious diseases by affecting the ecological environment of pathogens, exposure probability, and host susceptibility, thus resulting in the occurrence of the disease.

In the study, in order to analysis the spatial heterogeneity of the influence of meteorological factors on HFMD, the relationships between HFMD and meteorological factors was further calculated in three strata classified by the BSTHM in hot spots, cold spots and neither hot nor cold spots, respectively. The results indicated that there presented distinctive local relationships in each stratum compared with those in global model (Table 1, Additional file 1: Tables S2, S3 and S4). In the global model, average temperature and relative humidity were found to be key factors affecting HFMD risk, however it indicated no statistically significant influence of average temperature on HFMD risk in the cold spots (Additional file 1: Table S3), and the effect of relative humidity was also not found statistically significant relationship with HFMD risk in each stratum (Additional file 1: Tables S2, S3 and S4). The potential reasons for these difference between global and local models may be that there existed different HFMD transmission mechanisms in different regions, and small size of samples in each stratum also affected the statistically significant level of estimated parameters.

In addition, the study indicated that the distribution of HFMD risk presented apparently spatial heterogeneity. High risk of HFMD (hot spots) was mainly concentrated in the areas where the level of economic and urbanization was high, while low risk of HFMD (cold spots) was mainly distributed in the undeveloped counties having lower economic level and incomplete infrastructure [35], which was consistent with previous studies. For example, one previous study found that the proportion of tertiary industry was positively correlated to the incidence of HFMD [36]. One previous study found that incidence in economically developed areas, for example Beijing, Tianjin, Shanghai, and Zhejiang, are higher than in less developed areas [19]. Furthermore, a study found that population density and tertiary industry presented the most significant impact on this disease, explaining 42% of the HFMD transmission [54]. The potential mechanism may be that, due to the rapid economic development and urbanization in recent years, there exists increased floating population in the more developed regions compared with cold spots, however, there is limited living and work room, which providing more opportunities for contact between each other, thus accelerating the spread of the virus.

In the study, three models, GeoDetector, BSTHM and SLM, were used, in which the BSTHM is linear models used to detect the spatiotemporal heterogeneity of the HFMD risk. However, HFMD transmission in reality has a fundamentally non-linear nature, and a linear method was a first-order approximation for reality. This introduces some uncertainty to the results of the study. Fortunately, in a linear model, the physical mechanism of parameters is clear, and the calculation is easy to implement and repeat.

## Conclusions

The present study describes the detailed spatiotemporal dynamics of HFMD and its relationships with meteorological and socio-economic factors from 2012 to 2013 in Henan province, China. The high risks were mainly concentrated in regions where the level of economic was high. HFMD risk in Henan had an obviously seasonal characteristic, which indicated that HFMD risk is mainly related to a hot and humid environment. These results provide a good illustration for the spatiotemporal distribution and the seasonal variation of HFMD risk among different geographic areas, which can serve as reference and basis for the surveillance and control of this disease in practice.

## Additional file

Additional file 1. **Table S1.** contains descriptive characteristics for meteorological and socio-economic variables selected in this study. **Tables S2–S4.** contain the estimated the posterior means and RR of BSTHM coefficients in three strata. (DOCX 23 kb)

## Abbreviations

BSTHM: Bayesian space-time hierarchy model
BYM: Besag, York, and Mollie
CAR: Conditional autoregressive
GDP: Gross Domestic Product
HFMD: Hand, foot and mouth disease
MCMC: Markov chain Monte Carlo
SLM: Spatial lag model
