# Supplementary material for: Space-time heterogeneity of hand, foot and mouth disease in children and its potential driving factors in Henan, China
Source: BMC Infect Dis. 2018 Dec 7;18:638. doi: 10.1186/s12879-018-3546-2 (PMC6286567; doi:10.1186/s12879-018-3546-2)
Supplement: Supplementary file 1 — Table S1. contains descriptive characteristics for meteorological and socio-economic variables selected in this study. Tables S2–S4. contain the estimated the posterior means and RR of BSTHM coefficients in three strata. (DOCX 23 kb) [file 12879_2018_3546_MOESM1_ESM.docx]

Supporting information

Title:

Space-time Heterogeneity of Hand, Foot and Mouth Disease in Children and Its Potential Driving Factors in Henan, China

**Author names and affiliations:**

Xiangxue Zhang^#a,b^, Chengdong Xu^#*b^ , Gexin Xiao^c^

^a^The School of Earth Science and Resources, Chang’an University, Xi’an 710054, China

^b^State Key Laboratory of Resources and Environmental Information System, Institute of Geographic Sciences and Natural Resources Research, Chinese Academy of Sciences, Beijing 100101, China

^c^China National Center for Food Safety Risk Assessment, Beijing 100022, China

^*^Corresponding author at: State Key Laboratory of Resources and Environmental Information System, Institute of Geographic Sciences and Natural Resources Research, Chinese Academy of Sciences, 11A, Datun Road, Chaoyang District 100101, Beijing, China.

Email addresses of all author: [zxx@lreis.ac.cn](mailto:zxx@lreis.ac.cn) (X.Zhang); [xucd@lreis.ac.cn](mailto:xucd@lreis.ac.cn) (C.Xu); [biocomputer@126.com](mailto:biocomputer@126.com) (G.Xiao)

Email addresses of corresponding author: [xucd@lreis.ac.cn](mailto:xucd@lreis.ac.cn) (C.Xu)

^#^Contributed equally.

**Tables**

**Table S1** Descriptive characteristics for monthly meteorological and yearly socio-economic variables

**Table S2** The estimated posterior means and RR of coefficients in BSTHM of hot spots

**Table S3** The estimated posterior means and RR of coefficients in BSTHM of cold spots

**Table S4** The estimated posterior means and RR of coefficients in BSTHM of other spots

**Table S1** Descriptive characteristics for monthly meteorological and yearly socio-economic variables

| Variables | Minimum | 25% | Mean | SD | Median | 75% | Maximum |
| --- | --- | --- | --- | --- | --- | --- | --- |
| Mean temperature (°C) | -3.00 | 5.58 | 15.11 | 10.00 | 16.70 | 23.63 | 30.30 |
| Relative humidity (%) | 38.00 | 57.00 | 63.37 | 8.68 | 64.00 | 70.00 | 84.00 |
| Precipitation (mm) | 0.00 | 11.00 | 46.86 | 55.54 | 25.80 | 61.63 | 433.90 |
| Air pressure (hPa) | 921.00 | 990.00 | 997.47 | 19.17 | 1001.00 | 1012.00 | 1024.00 |
| Wind speed (m/s) | 0.80 | 1.70 | 1.90 | 0.43 | 2.00 | 2.10 | 3.60 |
| Sun hours (h) | 33.00 | 126.00 | 155.34 | 46.93 | 159.00 | 183.00 | 298.00 |
| Ratio of urban to rural population | 0.24 | 0.33 | 1.03 | 1.94 | 0.43 | 0.69 | 15.02 |
| Proportion of the tertiary industry (100%) | 8.70 | 21.20 | 26.69 | 8.41 | 25.80 | 30.40 | 64.70 |
| Per capita GDP (10^4^CNY) | 1.12 | 1.70 | 3.48 | 3.08 | 2.42 | 4.24 | 21.40 |
| Proportion of the second industry (100%) | 32.00 | 42.63 | 54.76 | 13.43 | 53.65 | 66.63 | 87.70 |
| High school enrollment rate (100%) | 25.50 | 37.13 | 46.23 | 14.13 | 44.70 | 51.68 | 134.40 |
| Per capita income of farmers (10^3^CNY) | 4.39 | 5.63 | 7.12 | 1.82 | 6.83 | 8.31 | 13.12 |
| Population density of children under five (10^4^person/km^2^) | 0.70 | 3.94 | 5.68 | 2.89 | 5.25 | 6.77 | 21.46 |

**Table S2** The estimated posterior means and RR of coefficients in BSTHM of hot spots

| Meteorological factors | Posterior mean (95% CI) (100%) | RR (95% CI) |
| --- | --- | --- |
| Average temperature (°C) | 5.20 (1.87-8.59) | 1.05 (1.02-1.09) |
| Relative humidity (%) | 1.58 (-0.23-3.41) | 1.02 (1.00-1.03) |
| Air pressure (hPa) | 1.04 (0.10-1.70) | 1.01 (1.00-1.02) |
| Precipitation (mm) | -0.18 (-0.47,0.11) | 1.00 (0.995-1.001) |
| Sun hour (h) | -0.01 (-0.42,0.40) | 1.00 (0.996,1.004) |
| Wind speed (m/s) | 15.51 (-11.15-42.38) | 1.17 (0.89-1.53) |

**Table S3** The estimated posterior means and RR of coefficients in BSTHM of cold spots

| Meteorological factors | Posterior mean (95% CI) (100%) | RR (95% CI) |
| --- | --- | --- |
| Average temperature (°C) | 2.28 (-1.69-6.40) | 1.02 (0.98-1.07) |
| Relative humidity (%) | 1.87 (-0.75-4.52) | 1.02 (0.99-1.05) |
| Air pressure (hPa) | 0.36 (-0.39-1.07) | 1.004 (1.00-1.01) |
| Precipitation (mm) | -0.20 (-0.40,0.01) | 0.998 (0.996-1.00) |
| Sun hour (h) | 0.01 (-0.52,0.53) | 1.00 (0.99,1.01) |
| Wind speed (m/s) | 44.19 (13.17-75.02) | 1.56 (1.14-2.12) |

**Table S4** The estimated posterior means and RR of coefficients in BSTHM of other spots

| Meteorological factors | Posterior mean (95% CI) (100%) | RR (95% CI) |
| --- | --- | --- |
| Average temperature (°C) | 4.60 (1.31-8.35) | 1.05 (1.01-1.09) |
| Relative humidity (%) | 0.01 (-1.36-1.39) | 1.00 (0.99-1.01) |
| Air pressure (hPa) | 1.36 (0.61-1.96) | 1.01 (1.006-1.02) |
| Precipitation (mm) | 0.02 (-0.12,0.16) | 1.00 (0.999-1.002) |
| Sun hour (h) | 0.08 (-0.21,0.36) | 1.00 (0.998,1.004) |
| Wind speed (m/s) | -27.27 (-45.72--8.77) | 0.76 (0.63-0.92) |
